# Kaempferol: Antimicrobial Properties, Sources, Clinical, and Traditional Applications

**DOI:** 10.3390/ijms232315054

**Published:** 2022-11-30

**Authors:** Argyrios Periferakis, Konstantinos Periferakis, Ioana Anca Badarau, Elena Madalina Petran, Delia Codruta Popa, Ana Caruntu, Raluca Simona Costache, Cristian Scheau, Constantin Caruntu, Daniel Octavian Costache

**Affiliations:** 1 Department of Physiology, The “Carol Davila” University of Medicine and Pharmacy, 050474 Bucharest, Romania; 2 Akadimia of Ancient Greek and Traditional Chinese Medicine, 16675 Athens, Greece; 3 Pan-Hellenic Organization of Educational Programs (P.O.E.P), 17236 Athens, Greece; 4 Orasis Acupuncture Institute, 11526 Athens, Greece; 5 Department of Biochemistry, The “Carol Davila” University of Medicine and Pharmacy, 050474 Bucharest, Romania; 6 Department of Toxicology, Grigore Alexandrescu Emergency Children’s Hospital, 011743 Bucharest, Romania; 7 Department of Hematology, Fundeni Clinical Institute, 022328 Bucharest, Romania; 8 Department of Oral and Maxillofacial Surgery, ‘Dr. Carol Davila’ Central Military Emergency Hospital, 010825 Bucharest, Romania; 9 Department of Oral and Maxillofacial Surgery, Faculty of Dental Medicine, ‘Titu Maiorescu’ University, 031593 Bucharest, Romania; 10 Department of Gastroenterology, Gastroenterology and Internal Medicine Clinic, ‘Dr. Carol Davila’ Central Military Emergency Hospital, 010825 Bucharest, Romania; 11 Department of Internal Medicine and Gastroenterology, ‘Carol Davila’ University of Medicine and Pharmacy, 050474 Bucharest, Romania; 12 Department of Dermatology, ‘Prof. N.C. Paulescu’ National Institute of Diabetes, Nutrition and Metabolic Diseases, 011233 Bucharest, Romania; 13 Department of Dermatology, ‘Dr. Carol Davila’ Central Military Emergency Hospital, 010825 Bucharest, Romania

**Keywords:** kaempferol, molecular mechanisms, antibacterial, antiprotozoal, antifungal, herbal medicine

## Abstract

Flavonoids are a category of plant-derived compounds which exhibit a large number of health-related effects. One of the most well-known and studied flavonoids is kaempferol, which can be found in a wide variety of herbs and plant families. Apart from their anticarcinogenic and anti-inflammatory effects, kaempferol and its associated compounds also exhibit antibacterial, antifungal, and antiprotozoal activities. The development of drugs and treatment schemes based on these compounds is becoming increasingly important in the face of emerging resistance of numerous pathogens as well as complex molecular interactions between various drug therapies. In addition, many of the kaempferol-containing plants are used in traditional systems all over the world for centuries to treat numerous conditions. Due to its variety of sources and associated compounds, some molecular mechanisms of kaempferol antimicrobial activity are well known while others are still under analysis. This paper thoroughly documents the vegetal and food sources of kaempferol as well as the most recent and significant studies regarding its antimicrobial applications.

## 1. Introduction

In general, natural substances have been a recent target of research for their numerous health benefits and also for their potential as the basis for new drugs [1,2,3]. The use of plants and herbs is documented by numerous authors both in Europe [4] and elsewhere [5,6,7,8]. The aim of such research is two-pronged, both to explore new opportunities for effective therapeutical agents, and also to elucidate the correlation between a decreased incidence of health problems and the consumption of certain food types. Regarding this last aim, it is the logical course of action, since certain diets are correlated with negative mortality and morbidity incidence rates [9,10,11,12,13]—in addition, based on the research of [14] specific diet choices after the diagnosis of cancer may improve survival rates.

The focus of this paper is kaempferol, (3,5,7-trihydroxy-2-(4-hydroxyphenyl)-4H-chromen-4-one), a flavonoid with many promising health benefits found in a variety of plants. Kaempferol is named in honor of Engelbert Kaempfer, a German doctor, naturalist, and historian who lived during the 17th century and made a significant contribution to transporting medical knowledge from Japan to the West [15]. Kaempferol, as a chemical compound, was discovered in *Camelia sinensis* (tea tree) [16] and exhibits a host of different positive health-related effects.

In this review, we will present a thorough view of the studies which have aimed to ascertain the use of kaempferol against pathogens, namely protozoa, fungi and bacteria, describing the molecular mechanisms of action, where literature data is available. We will also explain the relative importance of the pathogens described to justify the importance of the studies on kaempferol as a novel basis for therapies and drug design. A number of these researches have focused on the extracts of plants that are included in traditional medical systems in different countries and regions. Accordingly, we will also describe the traditional use of kaempferol-containing plants and we will also present the most prominent plant species which contain kaempferol in regard to biosynthesis and availability of the substance.

## 2. Biosynthesis and Availability of Kaempferol

Kaempferol is a flavonoid; flavonoids are regarded as the largest group of secondary plant metabolites. They are polyphenolic compounds of low molecular weight and are used by plants to stimulate and regulate their growth and for defense purposes [17]. Flavonoids are divided into a number of groups based on their chemical composition, namely flavones, flavonols, flavanones, isoflavonoids, neoflavonoids, catechins (flavanols), anthocyanins and chalcones [18]. The antioxidant properties of polyphenols—flavonoids are such compounds—are already well known [19]; more than 10^4^ types of flavonoids are estimated to exist [20,21]. Other proven effects of flavonoids include hepatoprotective [22,23,24], antimicrobial [25,26], renoprotective [27,28], antidiabetic [29,30], cardioprotective [31,32], anti-arthritic [33], neuroprotective [34,35,36,37], gastroprotective [38,39] and anti-mutagenic [40,41,42,43,44], among others [16].

Recently, there has been an increasing amount of research interest in the anti-carcinogenic potential of kaempferol [45,46], as a positive correlation between its consumption and reduced cancer incidence has been documented [47]; this is in addition to existing epidemiological studies linking increased flavonoid consumption with reduced cancer incidence [48,49]. The anti-inflammatory role of kaempferol has also been concisely presented by [50], while even its anti-adipogenic potential has come under investigation [51].

The basic structure of all flavonoids, regardless of their subclass, is a 15-carbon benopyranone or benzopyran in which the three-carbon bridge between the phenyl groups is commonly cyclized with oxygen forming a C6-C3-C6 flavan nucleus [19,52,53].

**Figure 1:**
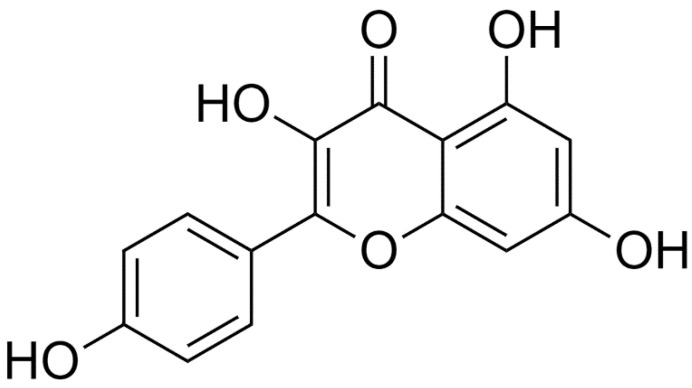
The two-dimensional structural formula of kaempferol.

Kaempferol is specifically classified as a flavonol [54] and has the molecular formula C_15_H_10_O_6_ (Figure 1).

### 2.1. Biosynthetic Pathways of Kaempferol

Flavonoids are synthetized via the shikimic acid pathway [55], a process that occurs in the plants’ plastids [56,57,58]. More than 2000 compounds are known, with nearly 500 occurring in a free-aglycone state and the rest as O- or C-glycosides. Flavonols, in their free forms as aglycones, have lipophilic properties, yet most flavonols produced in plants are attached to a sugar moiety, the glycoside form, and are water-soluble [59]. The hydroxyl functional groups present in each flavonol are potential sites for linkage to saccharides as O-glycosides [60]. The saccharides most commonly attached to flavonols are monosaccharides such as glucose, rhamnose, galactose, arabinose, and xylose [32], and the disaccharide rutinose (glucose and rhamnose connected by a β-glycosidic bond) [61].

### 2.2. Bioavailability of Kaempferol

The pharmacokinetics of kaempferol has been studied in vitro and in vivo, both in rats and humans. Flavonols such as kaempferol are commonly ingested as glycosides. The types and attachments of saccharide impact bioavailability, and also bioactivity [62].

Glycosides are highly polar compounds, a property that greatly impacts their absorption, whereas the intermediate polarity of aglycones facilitates it. For some types of glycosides, previous hydrolysis to absorbable aglycones is needed, and others can be absorbed without hydrolysis [54].

Like other flavonoids, kaempferol is mainly absorbed in the small intestine. The lipophilicity of aglycone kaempferol facilitates its absorption by passive diffusion, but evidence suggests that it can also be absorbed by facilitated diffusion or active transport [63]. The nature of sugar linking will influence the compound uptake, as enterocytes have a preference for glucose, as membrane-bound beta-glucosidase breaks down the glucoside before absorption [64]. Kaempferol glucosides with many sugar units in their structure travel to the large intestine, where gut microbiota will remove terminal saccharides exposing the glucose, and then absorption by enterocytes occurs [65]. Studies have shown that kaempferol-O-glycosides decomposition can be extended to a breakdown process known as C-ring fission (C-ring is the central ring of the flavonoid structure) to form simple phenolic compounds such as 4-hydroxyphenylacetic acid, phloroglucinol and 4- methylphenol, which can either be absorbed or excreted in feces [66,67,68].

After absorption, conjugated forms of kaempferol, some phenolic compounds produced by the colon microflora, kaempferol, and some kaempferol glycosides can reach systemic circulation and tissues and are transported along with intestinal metabolites to the liver, where a part of them are metabolized (mainly compounds with poor hydro solubility). In the liver, and also in enterocytes, metabolism involves the phase I (oxidation and O-demethylation) and the phase II pathway (sulfation, glucuronidation, and methylation) followed by distribution to body tissues and urine excretion [69,70,71]. 

Pure kaempferol can be isolated from several plant species (Table 1), in the Divisions Pteridophyta, Coniferophyta, and Angiosperms of the Kingdom Plantae [72]. In addition, the glycosides of kaempferol can be identified in many plant families [73,74,75,76,77,78,79,80]—a detailed account of the various kaempferol chemical compounds and derivatives may be found in recent studies [54]. The kaempferol content of some common foodstuffs is provided in Table 2.

## 3. Kaempferol as an Antibacterial Agent

The antibacterial properties of the secondary metabolites of plants have been in the foreground of research in the last two decades [189,190,191,192,193,194,195]. Such research is even more important considering the emergence of numerous resistant [196,197] and multi-drug resistant (MRD) bacteria [198]. Kaempferol-containing extracts and preparations, as well as pure kaempferol compounds, have been tested as possible antibacterials for quite some time [121,144,199].

The investigation into the action mechanisms behind the antibacterial activity of kaempferol has proven difficult due to the large variety within the family of kaempferol derivatives but also due to the diversity in morphology and functions between the numerous species of bacteria. However, some theories have been advanced and validated regarding the potential action mechanisms in specific bacteria. For instance, [200] have shown that a mixture of kaempferol 3-O-b-(200-acetyl) galactopyranoside and quercetin exerts antibacterial effects through cell membrane disruption, followed by activation of apoptosis and DNA fragmentation in *M. luteus* cells. Kaempferol was also the most effective tested flavonoid in damaging the cell membrane of *Escherichia coli* in a study by [201], where the findings were objectified by showing bacterial protein leakage into the extracellular environment. Moreover, kaempferol and quercetin interact with 3-oxyacyl-[acyl carrier protein] reductase (FabG) and enoyl-acyl carrier protein reductase therefore inhibiting the biosynthesis of fatty acids by *Mycobacterium*, *Pseudomonas aeruginosa*, and *Vibrio cholerae* thus hindering the function of the cell envelope as well as the impeding creation of bacterial biofilms [202,203,204]. Another important antibacterial mechanism was demonstrated for *E. coli*, where kaempferol was shown to be the most effective flavonoid in directly inhibiting the bacterial DNA gyrase [205]; similarly, kaempferol inhibited the DNA gyrase in methicillin-resistant *Staphylococcus aureus* [206]. Kaempferol was also able to inhibit DNA helicases, more specifically SAPriA in *Staphylococcus aureus*, as shown by [207].

Actions of kaempferol compounds against *Porphyromonas gingivalis*, *Prevotella intermedia,* and *Cutibacterium acnes* have been described by [144,208]. The research of [209] had already indicated the antibacterial effect of the extract of *S. hymettia* against *Enterobacter cloacae*, and also other bacteria, as will be presented below. The extract of *Helichrysum compactum*, which contained pure kaempferol and also kaempferol-3-O-glucoside, proved to have a degree of antibacterial activity [178]. It is also possible, that the extract from *Nephelium lappaceum*, which contains kaempferol compounds, has antimicrobial activity [210]. A local Malaysian herb, kacip Fatimah, i.e., the plant *Labisa pumila* Benth, which contains kaempferol, was found to have some antibacterial activity against *Micrococcus luteus*, *Bacillus subtillis*, *Bacillus cereus*, *Staphylococcus aureus*, *Enterobacter aerogenes*, *Klebsiella pneumoniae*, *Escherichia coli* and *Pseudomonas aeruginosa*, albeit at relatively low bacterial loads [211]. The extract of *Uapaca heudelotti* proved effective against *S. pneumoniae* [212], as well as against other pathogens. It is also important to note that while some kaempferol-containing extracts may not have significant antibacterial action on their own, they may potentiate the action of some antibiotics [213].

Subsequently, we will present the most important research on the antimicrobial activities of kaempferol against different bacterial genera (Table 3), which are human pathogens of particular interest.

### 3.1. Antibacterial Activity against Acinetobacter baumannii

This coccobacillus was once considered of low importance, from a medical standpoint, but has now emerged as a prominent healthcare unit-acquired and community-acquired infection. It frequently causes pulmonary infections and septicemia in immunocompromised patients [231]. Its antibiotic resistance and increased survival in harsh environments [232,233,234] further enhance its pathogenicity. At the moment, the results of the kaempferol-containing compounds against this pathogen are quite promising, and this can be important in the face of ever-increasing antibiotic resistance [96,215].

The compound kaempferol-3,7-O-α-l-dirhamnoside was found to be moderately inhibitory against *A. baumannii* [214]. A novel nanotechnology application involving a blend loaded with kaempferol nanocrystals showed very promising results against *A. baumannii* [215]; the research focused on treating infected wounds. Kaempferol-containing propolis extracts have also proved effective against *A. baumannii* in vitro [216]. The action of a further kaempferol-containing compound, the extract of *Geranium ibericum* subsp. *jubatum*, was also found to be almost as effective as some commercial antibiotics against this pathogen in vitro [132]. Earlier research [96] indicated that the kaempferol-containing extract of *K. fedtschenkoi* was effective against this pathogen.

### 3.2. Antibacterial Activity against Bacillus Spp.

In general, the bacteria of this genus are aerobic ([235]; and references therein), rod-shaped bacteria [236], which are spore-forming and resistant to disinfectants and harsh environmental conditions [236,237]. According to [238], only a handful of species from this genus are pathogenic; although current research has focused on the antibacterial actions of kaempferol against *B. subtilis*, which is non-pathogenic, the existing research experience can be potentially used in the future to find effective antimicrobial phytochemicals against the pathogenic bacillus species, namely *B. anthracis* and *B. cereus* [239,240]. 

An extract of taif’s rose (*Rosa damascena* Mill. var. trigintipetala) exhibited antimicrobial activity against *B. subtilis* as well as other microorganisms. The extract contained kaempferol amongst other compounds [218]. Earlier research by [214] indicated that the antibacterial action of kaempferol-3,7-O-α-l-dirhamnoside, on its own, was quite moderate. The fermented aerial part of *Bupleurum chinense* also contains kaempferol and exhibited promising antibacterial action against this bacterium [172]. The compound kaempferol-3-O-glucoside, which was isolated from the stem bark of *Uapaca heudelotti*, was also effective in that regard [212]. 

Based on the research of [167], the kaempferol compounds of the extract of *Buddleja indica* Lam. enable it to act as a local antiseptic, effective against *B. subtilis*. The novel research of [100] on the extract of *Astragalus creticus*, which, among other compounds, contains kaempferol and kaempferol-7-O-β-D-glucopyranose, proved their efficacy against this pathogen. Finally, the conjugation of kaempferol with silver nanoparticles proved effective against *B. subtilis* [217].

### 3.3. Antibacterial Activity against Escherichia coli

These bacteria are physiological colonizers of the gastrointestinal tract; the colonization begins typically shortly after birth. They typically do not cause disease in immunocompetent patients but will become pathogenetic, if they migrate to other locations or if their host becomes immunocompromised [241]. The most well-described *E. coli* pathogenic categories are the enteropathogenic *E. coli* (EPEC), the enterohaemorrhagic *E. coli* (EHEC), the enterotoxigenic *E. coli* (ETEC), the enteroaggregative *E. coli* (EAEC), the enteroinvasive *E. coli* (EIEC) and the diffusely adherent *E. coli* (DAEC) [242]. Commonly, *E. coli* infections are centered around the gastrointestinal and urogenital systems. Although most such infections can be easily treated, the emergence of multi-drug resistant (MDR) *E. coli* presents a novel therapeutical challenge [243].

The anti-microbial action of kaempferol-3,7-O-α-l-dirhamnoside was moderate against *E. coli* [214]. Success in that regard was also documented by [209] who used the extract of *S. hymettia*. The extract of *B. chinense*, which contained kaempferol-3-O-β-D-rutinoside and kaempferol proved effective against this bacterium [172]. The propolis extracts studied by [216] were also found to be effective against this pathogen. The combination of kaempferol with silver nanoparticles was also proven to be effective against *E. coli* [217]. The phytochemical extracts of [218,219] also proved effective against *E. coli*.

### 3.4. Antibacterial Activity against Klebsiella pneumoniae

*Klebsiella pneumoniae* represents an important human opportunistic pathogen and an emerging concern in clinical settings [244]. It accounts for virtually one-third of the total Gram-negative bacterial infections [245]. *Klebsiella* infections, especially in nosocomial settings, are rather severe [246]. The emergence of *K. pneumoniae* strains which are resistant to even last-line antibiotics [244,247] means that is not improbable, in the near future, that new compounds, whether natural or artificial, will be required to counter it. Interestingly, a strain of Klebsiella was found to even be resistant to chlorine treatment in water [248].

The anti-microbial action of kaempferol-3,7-O-α-l-dirhamnoside, was moderately effective against *K. pneumoniae* [214]. A similar anti-*Klebsiella* activity was also found by [172]. Earlier research by [220], on the extract of *Argyreia speciosa*, which was determined to contain kaempferol 7-O-methyl-3-sulphate, showed that it was inhibitory for *K. pneumoniae* growth. Similar successful antibacterial action was documented by [209], who used the extract of *S. hymettia*. The extract studied by [132] proved to also be effective against *K. pneumoniae*, as well as the extract studied by [219].

### 3.5. Antibacterial Activity against Mycobacterium Spp.

From the *Mycobacterium* genus, the most well-known and dangerous pathogen is *Mycobacterium tuberculosis*, which is the causative agent of tuberculosis, one of the oldest human diseases [249]. Although a vaccine against the disease exists, it is of varying efficiency [250] and has proven incapable of stopping the global epidemic [251]. While there exist antibiotics effective against tuberculosis during the last few years, the increase in antibiotic resistance of *M. tuberculosis* has led to the emergence of multi (MDR) [252], extensively (XDR), extremely (XXDR) and total (TDR) drug-resistant strains; these are estimated to kill about 75 · 10^6^ people, in the next three decades [253]. Although resistance-conferring mutations may reduce the overall fitness of the bacteria, it has been suggested by [254,255,256] that the resistant bacteria may find ways to circumvent this limitation. Thus, it is evident that tuberculosis may again come to the foreground as a major disease, even in Western countries. *M. bovis* infects primarily cattle but can also spread to humans [257,258,259,260]; however, it is not of particular importance as a human pathogen [261]. Rather, its study is of interest in understanding the pathogenetic mechanism of *M. tuberculosis* [262].

Based on the research of [230], a leaf and hardwood extract from *Vatairea macrocarpa*, a plant used in Brazilian folk medicine, exhibited antibacterial action, in an in vivo model, in rat paws infected with *M. bovis*. The action of kaempferol-3-O-rhamnopyranoside was supplemented by that of other flavonoids in the extract. The extract was also found to have significant anti-inflammatory parameters. 

The extract of *Argyreia speciosa* was found to have antibacterial properties against *M. tuberculosis* [220]. Another medicinal plant, *Doliocarpus dentatus*, proved to be effective in a rat model, as an antimycobacterial agent; the phenolic extract of its leaves contains kaempferol 3-O-α-L-rhamnopyranoside [229]. The extract of *Pluchea indica*, which contained kaempferol, was identified as a potent inhibitor of the *M. tuberculosis* CYP121 in a recent study by [180]. Finally, pure kaempferol from *Bauhinia vahlii*, was found, along with other flavonols, to be effective against *M. tuberculosis* [102].

### 3.6. Antibacterial Activity against Pseudomonas aeruginosa

This is a versatile opportunistic pathogen, from a metabolic point of view, which can cause both localized and systemic infections in humans, of varying degrees of severity [263]; recently, it has come to the foreground as a potent causative agent of nosocomial infections [264]. People already suffering from cystic fibrosis and COPD are at an increased risk of contracting *P. aeruginosa*, even outside of healthcare units [265,266,267]. It is of particular note that in cystic fibrosis patients, the bacterium may persist for decades [268]. Although some of the *P. aeruginosa* infections are relatively easily treated [269,270,271], other cases are still characterized by increased morbidity and mortality [267,272,273,274]. It has been observed that there is increasing resistance to antibiotics, of many *P. aeruginosa* strains, which is caused both by acquired and intrinsic mechanisms; this necessitates the development of new treatment avenues [275].

The research of [209], who tested the extract of *S. hymettia*, indicated that kaempferol-containing compounds were effective against *P. aeruginosa*. The extract of *Bryophyllum pinnatum* (Lank.) Oken also had some antibacterial activity against *P. aeruginosa* [221].

The extract prepared by [96] exhibited good antibacterial activity against *P. aeruginosa*. The extract of *Bupleurum chinense*, which has been already mentioned, proved effective against this pathogen [172]. The extract from *Y. gigantea*, which contains kaempferol-3-O-α-l-rhamnoside, was found to have an antimicrobial potential against this pathogen [219]. 

### 3.7. Antibacterial Activity against Salmonella Spp.

Salmonella is a common pathology in both developed and developing countries and represents a major public concern [276,277]; there are over 2600 recorded serotypes [278]. Salmonellae are foodborne pathogens, found mostly in poultry, eggs, and dairy products [279]. Recently, there has been an increase in the number of antibiotic-resistant strains; these are strains of increased virulence that are associated with increased mortality [280].

The extract of *Uapaca heudelotti* was effective, as an antimicrobial, against *S. typhi* [212]. Another effective antimicrobial against this pathogen is the extract of *Bryophyllum pinnatum* (Lank.) Oken [221]. The extract from *Yucca gigantea* also had an effect against *S. typhimurium* [219].

### 3.8. Antibacterial Activity against Staphylococcus Spp.

*Staphylococcus aureus* is a frequent human commensal and a common cause of various infections in humans. It can cause a wide variety of pathologies and associated symptoms, ranging from skin and soft tissue infections to infective endocarditis [281]; different staphylococcal strains are characterized by different aggressiveness properties [282]. The importance of *S. aureus* as a pathogen is further highlighted by the emergence of increasing antibiotic resistance [283,284]. The particular strain of *Staphylococcus aureus* which is resistant to methicillin is commonly referred to as MRSA (methicillin-resistant *Staphylococcus aureus*); it is a significant problem for health systems worldwide, both from a medical and a healthcare cost standpoint [285,286]. Its incidence rates present significant variations depending on the countries and healthcare unit location but are nevertheless quite significant [287,288]. The situation is aggravated even more since different types of MRSA have been identified, namely the healthcare-associated MRSA (HA-MRSA), the community-associated MRSA (CA-MRSA), and the livestock-acquired MRSA (LA-MRSA) [289]. A rather more benign species is *S. epidermidis*, a commensal which is not a frequent cause of disease, but it is of increasing importance in nosocomial settings; in healthcare unit settings, its infection rates are approximately commensurate with those of *S. aureus* [290].

The early research of [214] indicated that kaempferol-3,7-O-α-l-dirhamnoside was particularly effective against *S. aureus*. The research of [209], on the extract of *Scabiosa hymettia*, which contained two kaempferol-based flavonoids, corroborated the antibacterial action of kaempferol and its derivatives, against *S. aureus*; it was also active against *S. epidermidis*. The extract of *M. scaber*, a plant used in traditional West African medicine, also proved effective against *S. aureus* [223]. It must be noted that in this last case, when the compounds of the extract were tested separately, kaempferol-3-O-rutinoside exhibited a low antibacterial action suggesting that either the antibacterial effects were attributable to other compounds or that it has some sort of synergistic action with some of the other compounds found in the extract. A degree of antibacterial activity, against *S. aureus*, was exhibited by some of the extracts of *Allium ursinum* from Bulgaria [224]. The extract of *Bryophyllum pinnatum* (Lank.) Oken also exhibited interesting antibacterial properties against *S. aureus* [221].

The experiments of [222] determined that both kaempferol 3-O-α-L-(2″,4″-di-E-p-coumaroyl)-rhamnoside (C2) and kaempferol 3-O-α L-(2″-Z-p-coumaroyl-4″-E-p-coumaroyl)-rhamnoside (C3), exerted a strong antibacterial activity against different MRSA strains in vitro. These compounds were extracted from *Laurus nobilis*, and were virtually ineffective against *Streptococcus pneumoniae*, *Pseudomonas aeruginosa,* and *Serratia marcescens*. These same compounds were later found to have a synergistic effect with fluoroquinolones; namely, they increased the minimum inhibitory concentrations of these antibiotics. The same does not apply to hydrophobic quinolones [206]. 

Contemporary research [225], studying the anti-MRSA activities of the extract from *Platanus occidentalis*, determined that the numerous contained kaempferol compounds exhibited a satisfactory level of anti-MRSA activity. The research of [227], expounding upon the previous data, identified four isomers of kaempferol-3-O-α-L-(2”,3”-di-p-coumaroyl)-rhamnoside, from the same plant, which all exhibit anti-MRSA activity. It is possible that the main effect of such kaempferol-containing extracts is mostly attributable to the inhibition of the synthesis of the staphylococcal proteins, as determined by [228]. The earlier research of [226] also identified kaempferol-3-O-(2″,3″,4″-tri-O-galloyl)-α-l-rhamnopyranoside, along with other compounds, in the extract of *Calliandra tergemina* (L.) Benth. The anti-MRSA activity of the extract was verified experimentally. 

The already mentioned research of [215] indicated that kaempferol, in the form of nanocrystals, was effective against multi-drug resistant (MDR) *S. aureus*. Two different propolis extracts, which contained kaempferol, also proved effective against *S. aureus* [216]. The earlier research of [96] also indicated the effectiveness of a kaempferol-containing extract, against *S. aureus*. Another extract, from the plant *B. chinense* also proved effective against *S. aureus* [172]; the results from the extract of *Uapaca heudelotti* against *S. aureus* were also positive [212]. The combination of kaempferol with Ag nanoparticles was also effective against this pathogen [217]. A successful result was also obtained by [219], who studied the effects of the extract of *Y. gigantea*; this extract was effective against *S. epidermidis* too.

### 3.9. Antibacterial Activity against Enterococci

In the last decades, enterococci have become a concern as nosocomial pathogens of note [291]; they have the potential to cause serious infections [292,293]. The most important pathogens of the genus are *Enterococcus faecium* and *Enterococcus faecalis* [294]. In particular, vancomycin-resistant enterococci (VRE) present a serious challenge because not only can they resist many antibiotics but they are quick to accrue further resistance [295].

Based on the research of [214], kaempferol-3,7-O-α-l-dirhamnoside was quite effective against *Enterococcus faecalis*. The extract of *Laurus nobilis*, of [222], was also effective against VRE. The team of [140] isolated kaempferol, amongst other compounds, from the plant *Combretum erythrophyllum* and found that it was effective, as an antibacterial, against *E. faecalis*. 

### 3.10. Antibacterial Activity against Proteus Spp.

Perhaps the most important representative of the infectious species of the genus Proteus is *P. mirabilis*, which causes infections of the urinary tract, such as cystitis and pyelonephritis; many cases of asymptomatic bacteriuria have been also documented, predominantly in elderly patients and individuals having type 2 diabetes [296,297]. Such infections are also associated with urinary stone formation and even become life-threatening [298]. *P. vulgaris* has also been implicated in resistant healthcare unit-acquired infections [299]. Proteus infections can lead to catheter obstruction in catheterized patients [300] and the urinary stones created may act as a focal point for further bacterial infections [301]; indeed, catheterization is perhaps the dominant risk factor in Proteus infections [302,303]. The bacteria of this genus are associated with numerous determinants of antibiotic resistance [304,305] and there is even a number of MDR Proteus strains [306,307,308,309]; the prevalence of such strains was recently estimated to be quite high, at least in certain settings [310]. 

The already-mentioned extract of [218] was effective against *P. vulgaris*. Of all the microorganisms tested in this study, *P. vulgaris* proved to be the most susceptible. On the other hand, *Proteus mirabilis* proved quite resistant to kaempferol-3,7-O-α-l-dirhamnoside [214]. However, the extract studied by [132] was effective against this species, as well as the extract of *Uapaca heudelotti* [212]. This bacterial species proved also susceptible to the extract of *Y. gigantea*, which contains kaempferol-3-O-α-l-rhamnoside [219].

### 3.11. Antibacterial Activity against Vibrio cholerae

Cholera is most certainly an ancient disease of humans, although it has become a major health concern after the 19th century; it is a physiological inhabitant of aquatic ecosystems [311,312,313]. There is a number of pathogenic biotypes and there are several virulence factors [314]. The emergence of resistant strains of *Vibrio cholerae* has been documented in the past [315] and further resistance mechanisms continue to be observed [316].

Kaempferol and some of its derivatives were found to be effective against *Vibrio cholerae*, showing good antibacterial activity; in particular, kaempferol did not exhibit side effects such as toxicity to lymphocytes [140].

## 4. Antifungal Properties of Kaempferol

A very small number of fungi species are pathogenic to humans [317]; of these pathogenic fungi, some cause mild infections, while others, such as *Candida* spp. and *Aspergillus* spp., can even cause life-threatening, systemic infections [318]. Based on recent research, infections by *Candida* species, in hospital settings, represent an increasing health problem [319,320]. While fungi of this genus are generally benign, they can be the cause of oral candidiasis; in women, a significant percentage will suffer, sometime in their lives, from vaginal candidiasis [321,322]. Infections by *Candida* species are mostly determined by risk factors [323,324]. 

Likewise, *Aspergillus fumigatus*, while harmless to the immunocompetent host, will cause aspergillosis in immunocompromised patients; this represents an increasing concern with the number of such patients rising [325,326,327,328]. It is important to note that it is virtually impossible to evade exposure to this pathogen, as humans ingest hundreds of its conidia on a daily basis [329,330,331]. Another important aspect of *A. fumigatus* infections is that they may occur in the cavities left over in patients who have recovered from tuberculosis [332,333]; this is interesting, considering that kaempferol is known to be effective against *M. tuberculosis*, as already discussed. Therefore, for this specific case, a kaempferol-containing agent could both suppress the initial infection and act preventatively against possible aspergillosis. 

Finally, *Cryptococcus neoformans* is one of the deadliest fungal pathogens [334] and, according to a recent survey kills thousands of infected patients each year [335]. Its importance as a disease of global interest was realized after the 1970s [336,337]. Several risk factors are associated with an increased risk of cryptococcosis infection [338]; for example, as happens with other pathogens, cryptococcosis is particularly dangerous for HIV/AIDS patients [339]. In general, despite the availability of antifungal drugs, there is an emerging resistance as the microorganisms adapt [340]; coupled with the known side effects of many antifungal drugs [341], the importance of the development of novel therapeutic strategies, based on natural compounds becomes all the more obvious. 

Probably the first antifungal action of a kaempferol-containing compound was documented by [223], who tested the extract of *Mitracarpus scaber*, a plant used in traditional West African medicine. Both isolated kaempferol-3-O-[3-O-acetyl-6-O-(E)-p-coumaroyl]-b-d-glucopyranoside and kaempferol 3-O-b-D-kaempferol 3-O-b-D-glucopyranoside, from *S. hymettia*, were found to be active in vitro against *C. albicans*, *C. glabrata* and *C. tropicalis* [209]. As mentioned above, the kaempferol compound of the extract was not as effective when tested in isolated form. Moderate antifungal activity, against *C. albicans* was exhibited by some kaempferol-containing extracts from *Allium ursinum* [224]. The extract from *Labisa pumila* Benth, discussed before, also has a quite notable antifungal effect [211]. The extract from *Bryophyllum pinnatum* (Lank.) Oken exhibited interesting antifungal activity against *C. albicans*, *C. parapsilosis* and also *Cryptococcus neoformans* [221].

A comparatively weak inhibitory activity, at maximum concentration, was exhibited by kaempferol-3-O-(6”-galloyl)-β-D-glucopyranoside, isolated from *Baseonema acuminatum* [342]. Pure kaempferol also proved very effective against *C. albicans* both in vitro and in vivo in mice [343]. A possible effect against fungi of the *Candida* spp. was reported for the extract of *Trachyspermum ammi*, which contained kaempferol-(coumaroyl glucosyl)-rhamnoside [344]. The extract from *Y. gigantea* had a definite antifungal effect [219]. In the recent research of [345], it was determined, based on binding mechanisms, that kaempferol, at least when contained in an extract, may have a significant fungicidal effect in cases of vaginal candidiasis. 

The antifungal activity of kaempferol was also proven by the research of [132]. The extract prepared by [218], which also contained many other phytochemicals, had antifungal activity against *Candida albicans* and *Aspergillus fumigatus*. Moreover, significant fungistatic activity was exhibited by the bark extract of *Spondias mombin* [213]. A summary of the research on the antifungal properties of kaempferol mentioned in the text is presented in Table 4.

## 5. Antiprotozoal Properties of Kaempferol

Plant extracts containing kaempferol have shown antiprotozoal activity based on a number of researches against some of the most common protozoal pathogens (Table 5). In this section, we will review the most prominent research and list the plants identified as possible sources of cure and prevention (Table 6).

### 5.1. Antiprotozoal Action against Entamoeba histolytica and Giardia lamblia

Probably the first description of an antiprotozoal activity of a kaempferol-containing extract was made by [136] who studied the extract of *Helianthemum glomeratum* against *Entamoeba histolytica* in vitro, with successful results. Shortly after, kaempferol was also isolated from the roots of *Cuphea pinetorum*; it too was effective against *E. histolytica* and *Giardia lamblia* [356,357] in vitro. The importance of kaempferol in the antiprotozoal activity of these extracts was verified by [141]. The antiprotozoal activity of *H. glomeratum*, and the importance of kaempferol, were also examined by [358]. These plants are used in Mayan traditional medicine. The same promising results against *E. histolytica* were obtained when using the extract of *Morinda morindoides* [160]. The potency of kaempferol against *E. histolytica* was also demonstrated by [85]. Against both *E. histolytica* and *G. lamblia*, kaempferol-3,7-dimethylether was shown to have a degree of antiprotozoal activity in vitro [363]. This antiprotozoal activity of kaempferol against *E. histolytica* is important in the wider context of the activity of numerous flavonoids against this parasite [367]; such natural compounds may enable the development of new drugs against resistant parasites.

### 5.2. Antiprotozoal Action against Trypanosoma Spp.

This disease, also known as the sleeping sickness, is caused by *Trypanosoma brucei gambiense* and *Trypanosoma brucei rhodesiense*. It is a disease endemic to African countries [368]. It is mainly transmitted by flies of the genus *Glossina*, although transmission by other blood-sucking insects has also been documented [369,370]; it has even been proposed that due to the different possibilities of transmission, there may be outbreaks of this disease in non-endemic areas [371]. The treatment of this disease is based on a few drugs, which can be divided into two groups, the blood–brain barrier-crossing drugs (melarsoprol, eflornithine, nifurtimox) and the non-blood–brain barrier-crossing drugs (pentamidine, suramin) [372]. While currently there is a decrease in human African trypanosomiasis cases [373]. At the moment, resistance to treatment is not a massive issue for this particular disease, although resistant cases have been clinically reported [374]; furthermore, effective treatment options are required for the final stages of the disease [374].

Kaempferol-7-methylether was one of the compounds identified in the extract of *Alomia myriadenia* which was very effective against *Trypanosoma cruzi* in vitro [359]. On the other hand, the kaempferol-containing extract of *Conyza filaginoides*, was not found to be effective against *Trypanosoma* spp. and *Giardia* spp. [375]. Possibly, kaempferol is also important in the antiprotozoal activity exhibited by the bark extract of *Cayratia trifolia* Linn [98]. Contrary to that, the results of [376] were disappointing in that regard. Another research, focusing on kaempferol-3-O-methylether-5-O-β-D-glucoside and kaempferol-8-hydroxy-3,7-O-dimethylether-5-O-β-D-glucoside, from the extract of the plant *Zanthoxylum pistaciifolium* Griseb. found that they had no significant activity against either *T. cruzi* or *T. brucei* [377]. The compound 4′-methoxykaempferol, isolated from the extract of temperate propolis, proved to be quite effective against *T. brucei* [365]. The extract of *Lotus corniculatus* L. was found to be effective against *Trypanosoma* spp. [366].

### 5.3. Antiprotozoal Action against Plasmidium Spp.

Malaria is a well-known disease since ancient times and is caused by the amoeboid intracellular parasite Plasmodium; five of the 172 Plasmodium species are infectious to humans (*P. malariae*, *P.falciparum*, *P.vivax*, *P.ovale*, *P.knowlesi*); others are rarely infectious [378,379,380]. Regardless, their morphology and biology are quite similar [381]. The transmission of malaria is performed through its vectors, the female mosquitoes of the genus *Anopheles* [382]; subsequently, the parasite will infect first the hepatocytes and then the erythrocytes [383]. Currently, the most widespread therapy against malaria is the use of artemisinin and artemisinin-based combination therapy (ACT) [384]. In addition, there is an emerging resistance to antimalarial drugs, which threatens future efforts to eliminate the disease [385]. While endemic malaria is a major health concern, it may even be a health hazard in non-endemic countries [386].

Interestingly, 8-(1;1)-DMA-kaempferide, a flavonoid very similar to kaempferol [387], was found to have an antiprotozoal potential against *Plasmidium falciparum* [361]. Based on the research of [179], the extract of *Eupatorium perfoliatum* L. exhibited an in vitro antiprotozoal activity against *P. falciparum*; the extract contained kaempferol but the dimeric guaianolide was shown to be the most important part of the antiprotozoal activity. Some kaempferol metabolites proved to be effective against the malaria parasite when isolated in vitro [362]. The inability of kaempferol to influence negatively the formation of hemozoin, lead [388] to suggest that the in vitro antiplasmodial activity of kaempferol must not be related to any heme-binding activity pathway. The aforementioned study of [377], found two kaempferol glycosides to be ineffective against *P. falciparum*. Finally, the extract of *Lotus corniculatus* L., which contains pure kaempferol alongside some other kaempferol compounds has antiprotozoal activity against *Plasmodium* spp. [366].

### 5.4. Antiprotozoal Action against Leishmania Spp.

Leishmaniasis is a tropical and subtropical disease, mainly transmitted to humans through the sand flies of the genuses *Phlebotomus* and *Lutzomyia* [389]. The disease is extremely dangerous and presents a variety of symptoms; occasionally it can be fatal [390]. There exist over 20 species of the *Leishmania* parasite which can infect humans; leishmaniasis is a zoonosis and can be divided into visceral, cutaneous, and mucocutaneous [391]. The traditional treatment for leishmaniasis is based on antimonials, against which there is, however, increasing resistance [392]; antimonials also have frequent and rather severe side effects [393,394].

Compounds from the extract of *K. pinatta* were found to have antileishmanial activity [360]. In an in vitro assay, kaempferol-3,7-di-O-methylether was found to be able to induce cell death in *Leishmania amazonensis* [364]. When isolated from temperate propolis, 4′,7-dimethoxykaempferol was found to be quite effective against *L. amazonensis* in vitro [365].

## 6. Kaempferol-Containing Plants in Traditional Medical Systems

As mentioned elsewhere in this paper, a number of plants that contain kaempferol compounds are included in many traditional medical systems, all around the world. It is interesting to note that their traditional applications frequently correspond with their current effects under research. In this section, we will group the most important such plants, and their applications mentioned in this paper (Table 7), and then briefly examine the importance of some kaempferol-containing plants in the context of traditional Chinese medicine.

### Kaempferol-Containing Plants in the Context of Traditional Chinese Medicine

Herbal medicine is still regarded as an integral part of Traditional Chinese Medicine [437], and continues to be relevant in all parts of the world. As has been already proven, the study of these ancient practices can lead to novel therapies and drug discovery [438].

The use of the flower of the clove, known as dingxiang, is indicated to counteract the invasion of cold, and also to warm the kidneys; associated clinical signs include vomiting, hiccup, diarrhea, impotence, and leg weakness [439].

*Bupleurum chinense*, also known as *radix bupleuri*, or chaixu, is used in a variety of herbal formulas, which are associated with harmonizing lesser yang-stage disorders; some formulas, are also used against malaria. Other formulas, containing *B. chinense* are used to release exterior wind and heat [440]. To be more precise, *radix bupleuri* is derived from the roots of *B. chinense* [441]. Modern phytochemical research indicates that it has a wide range of pharmacological effects [442,443,444,445,446,447].

Geranium is also used in Traditional Chinese Medicine, both in anti-inflammatory and anti-microorganism applications [448]; current research has verified its anti-inflammatory potential [449].

*Astragalus creticus* is a plant that is endemic both to Greece and China [450]; it is used, either alone or in herbal formulas to warm the meridians and dispel cold [440]. In warming the meridians, it is ideal for rectifying the deficiency of the lung, spleen, and stomach meridians [439]. In Western Medicine terminology, it is used in cases of body weakness, as a diuretic, against digestive system disorders, or simply as a food supplement [451]. This is one of the most widespread plant genuses, and it has numerous ethnobotanical applications [452,453,454,455,456,457,458]. It would be interesting to compare the similarities between the applications of these plants in different medical systems.

Lastly, propolis, which is a bee product containing plant elements, not a plant per se, is an integral compound of many medicinal systems. It is used in traditional Chinese medicine, for its anti-inflammatory properties [459] and also has an anti-diabetic potential [460].

## 7. Discussion and Conclusions

In recent years, the field of phytochemistry has been rapidly developing with the aim of developing new drugs based on plant-derived compounds. At the same time, the field of ethnopharmacology studies the use of traditional medicinal plants of different regions and their possible applications in modern medical and pharmacological practice. Such approaches are integrated into the innovative practices which constitute the driving force behind the development of new therapeutical approaches [461].

As discussed in this paper, plants that contain kaempferol and its associated compounds have been tested for a number of effects, from anticarcinogenic to antibacterial, antifungal, and antiprotozoal. Indeed, the identification of natural compounds with anticarcinogenic potential has been a mainstay of medical research in the last decades [462]. Applications of such products have been proposed by [463] and the related new perspectives in drug discovery of many such natural agents have been summarized by [464]. 

Regarding the focus of this paper, in light of the promising effects of kaempferol compounds in the field of clinical microbiology, it can be said with a degree of certainty that it represents a novel potential for drug design. This is all the more important given the emerging resistance of many pathogens to traditional drugs. We may further postulate that given the wide range of kaempferol effects, drugs that may combat more than one condition may be developed; for example, using kaempferol as the basic agent, infections in cancer patients may be treated, combatting the pathogen and the cancer cells at the same time. This is a subject for future research.

Finally, as presented in the last part of the paper, kaempferol-containing plants are found in the traditional medicinal systems of almost every region; this attests to the efficacy of such treatments. In the particular case of traditional Chinese medicine, more often than not, such plants are used in conjunction with other plants and herbs, in herbal formulas. We propose, from a future research perspective, that these formulas should be tested, initially in vitro, to ascertain the relative efficacy of their components, and whether the kaempferol compounds of the ingredients can exert their actions on their own or in tandem with some of the other contained compounds.

## Figures and Tables

**Table 1:** Plant species containing pure kaempferol [54,81,82,83].

Clade	Order	Family	Species	Reference
Gymnosperms, Tracheophytes	Pinales	Taxaceae	*Taxus baccata*	[77]
Mesangiosperms, Magnoliids	Canellales	Canellaceae	*Warburgia stuhlmannii*	[84]
	Magnoliales	Annonaceae	*Annona cherimola* Miller	[85]
Mesangiosperms, Monocots	Asparagales	Alliaceae	*Allium cepa*	[86]
		Asphodelaceae	*Aloe vera*	[87]
	Liliales	Liliaceae	*Lilium candidum*	[88]
			*Lilium longiflorum*	[89]
		Smilacaceae	*Smilax bockii*	[90]
Mesangiosperms, Eudicots	Ranunculales	Berberidaceae	*Dysosma versipellis*	[91]
		Ranunculaceae	*Consolida oliveriana*	[92]
	Saxifragales	Crassulaceae	*Orostachys japonicus*	[93]
			*Rhodiola rosea*	[94]
			*Rhodiola sachalinensis*	[95]
			*Kalanchoe fedtschenkoi*	[96]
	Vitales	Vitaceae	*Parthenocissus tricuspidata*	[97]
			*Cayratia trifolia Linn*	[98]
	Cucurbitales	Cucurbitaceae	*Gynostemma cardiospermum*	[99]
	Fabales	Fabaceae	*Astragalus creticus* Lam.	[100]
			*Tylosema esculentum*	[101]
			*Bauhinia vahlii*	[102]
			*Acacia nilotica*	[103]
			*Amburana cearensis*	[104]
			*Cassia angustifolia*	[105]
			*Cassia siamea*	[106]
			*Indigofera suffruticosa*	[107]
			*Indigofera truxillensis*	[107]
			*Oxytropis falcate*	[108]
			*Securigera securidaca*	[109]
			*Tadehagi triquetrum*	[110]
			*Trifolium alexandrinum*	[111]
	Rosales	Elaeagnaceae	*Hippophae rhamnoides*	[112]
		Rhamnaceae	*Rhamnus nakaharai*	[113]
			*Rhamnus procumbens*	[114]
		Rosaceae	*Prunus davidiana*	[115]
			*Rosa* spp.	[116]
			*Rosa damascena*	[117]
			*Rosa hybrids*	[118]
		Ulmaceae	*Zelkova oregoniana*	[119]
	Celastrales	Celastraceae	*Euonymus alatus*	[120]
	Malpighiales	Clusiaceae	*Hypericum brasiliense*	[121]
			*Hypericum perforatum*	[122]
			*Vismia laurentii*	[123]
		Euphorbiaceae	*Elateriospermum tapos*	[124]
			*Euphorbia aleppica*	[125]
			*Phyllanthus acidus*	[126]
			*Sauropus androgynus*	[127]
			*Sebastiania brasiliensis*	[128]
		Salicaceae	*Populus davidiana*	[129]
	Geraniales	Geraniaceae	*Geranium carolinianum*	[130]
			*Geranium potentillaefolium*	[131]
			*G. ibericum* subs. *jubatum*	[132]
			*Pelargonium quercifolium*	[133]
	Brassicales	Brassicaceae	*Brassica rapa*	[134]
			*Bunias orientalis*	[135]
			*Diplotaxis erucoides*	[135]
			*Diplotaxis tenuifolia*	[135]
	Malvales	Cistaceae	*Helianthemum glomeratum*	[136]
		Malvaceae	*Althaea rosea*	[137]
		Sterculiaceae	*Theobroma grandiflorum*	[138]
		Tiliaceae	*Tilia tomentosa*	[139]
	Myrtales	Combretaceae	*Combretum erythrophyllum*	[140]
		Lythraceae	*Cuphea pinetorum*	[141]
		Myrtaceae	*Eucalyptus* spp.	[142]
			*Psidium guajava*	[143]
			*Syzygium aromaticum*	[144]
		Punicaceae	*Punica granatum*	[145]
	Sapindales	Anacardiaceae	*Pistacia vera*	[146]
			*Rhus verniciflua*	[147]
		Sapindaceae	*Koelreuteria henryi*	[148]
			*Koelreuteria paniculata*	[149]
		Simaroubaceae	*Simarouba versicolor*	[150]
	Caryophyllales	Amaranthaceae	*Alternanthera tenella*	[151]
		Nepenthaceae	*Nepenthes gracilis*	[152]
		Polygonaceae	*Polygonum tinctorium*	[153]
	Santalales	Santalaceae	*Thesium chinense*	[154]
	Ericales	Ebenaceae	*Diospyros lotus*	[155]
		Lecythidaceae	*Planchonia grandis*	[156]
		Myrsinoideae	*Ardisia colorata*	[157]
	Gentianales	Apocynaceae	*Echites hirsuta*	[158]
		Rubiaceae	*Morinda citrifolia*	[159]
			*Morinda morindoides*	[160]
	Vahliales	Vahliaceae	*Vahlia capensis*	[161]
	Solanales	Convolvulaceae	*Cuscuta australis*	[162]
			*Cuscuta chinensis*	[163]
		Solanaceae	*Solanum nigrum*	[164]
	Lamiales	Oleaceae	*Chionanthus retusus*	[165]
			*Olea europaea*	[166]
		Scrophulariaceae	*Buddleja indica* Lam.	[167]
		Lamiaceae	*Origanum dictamnus*	[168]
			*Rosmarinus officinalis*	[169]
	Apiales	Apiaceae	*Bunium persicum*	[170]
			*Bupleurum flavum*	[171]
			*Bupleurum chinense*	[172]
	Asterales	Asteraceae	*Heterotheca inuloides*	[173]
			*Chromolaena moritziana*	[174]
			*Ixeridium gracile*	[175]
			*Lactuca scariola*	[176]
			*Solidago virga-aurea*	[177]
			*Helichrysum compactum*	[178]
			*Europatorium perfoliatum* L.	[179]
			*Pluchea indica*	[180]
	Dipsacales	Caprifoliaceae	*Sambucus nigra*	[181]
Polypodiopsida, Moniliformopses	Polypodiales	Dennstaedtiaceae	*Dennstaedtia scabra*	[182]
Polypodiopsida, Ophioglossidae	Ophioglossales	Ophioglossaceae	*Ophioglossum petiolatum*	[183]

**Table 2:** Kaempferol content of some common foodstuffs (fresh unless otherwise specified).

Food	Quantity (mg/kg)	Reference
Capers	2590	[184]
Saffron	2050	[184]
Onion leaves	832	[185]
Arugula	590	[184]
Kale	470	[184]
Brown mustard	380	[184]
Pumpkin	371	[186]
Ginger	340	[184]
Cauliflower	270	[187]
Common beans	260	[184]
Carrot	140	[186]
Black tea	118	[186]
Chive	100	[184]
Endive	100	[184]
Collard	90	[184]
Broccoli	80	[184]
Fennel leaves	70	[184]
Goji berry (dried)	60	[184]
Green chilli	39	[186]
Strawberry	5–8	[188]

**Table 3:** Kaempferol compounds and their antibacterial activities based on current research.

Genus	Species	Tested Substance	MIC (μg/mL)	Year of Research	Reference
Gram-Negative Bacteria					
Acinetobacter	*A. baumannii*	Pure kaempferol-3,7-O-α-L-dirhamnoside	8	2006	[214]
		Extract from *Kalanchoe fedtschenkoi*	128–256	2019	[96]
		Artificial blend with nanocrystals	n/a—no adm. of sole kaempferol	2021	[215]
		Extract from *Geranium ibericum* subsp. *jubatum*	400	2021	[132]
		Extracts from propolis	n/a—expressed as % of propolis	2021	[216]
Enterobacter	*E. cloacae*, *E. aerogenes*	Extract from *Scabiosa hymettia*	n/a (only inh. zone data)	2008	[209]
		Extract from *Labisa pumila* Benth	Various depending on extract	2011	[211]
Escherichia	*E. coli*	Pure kaempferol-3,7-O-α-L-dirhamnoside	2	2006	[214]
		Extract from *Scabiosa hymettia*	n/a (only inh. zone data)	2008	[209]
		Extract from *Labisa pumila* Benth	Various depending on extract	2011	[211]
		Extract from *Bupleurum chinense*	n/a	2020	[172]
		Extracts from propolis	n/a—expressed as % of propolis	2021	[216]
		Conjugation of pure kaempferol with Ag nanoparticles	62.5	2021	[217]
		Extract from *Rosa damascena* Mill var. *trigintipetala*	n/a (only inh. zone data)	2022	[218]
		Extract from *Yucca gigantea*	13.3	2022	[219]
Klebsiella	*K. pneumoniae*	Pure kaempferol-3,7-O-α-L-dirhamnoside	4	2006	[214]
		Extract from *Scabiosa hymettia*	n/a (only inh. zone data)	2008	[209]
		Extract from *Argyreia speciosa*	2	2009	[220]
		Extract from *Labisa pumila* Benth	Various depending on extract	2011	[211]
		Extract from *Bupleurum chinense*	n/a	2020	[172]
		Extract from *Geranium* *ibericum* subsp. *jubatum*	400	2021	[132]
		Extract from *Yucca gigantea*	12.5	2022	[219]
Porphyromonas	*P. gingivalis*	Extract from *Syzygium aromaticum*	20	1996	[144]
Prevotella	*P. intermedia*	Extract from *Syzygium aromaticum*	20	1996	[144]
Proteus	*P. mirabilis*, *P. vulgaris*	Extract from *Uapaca heudelotti*	2	2020	[212]
		Extract from *Geranium* *ibericum* subsp. *jubatum*	300	2021	[132]
		Extract from *Rosa damascena* Mill var. *trigintipetala*	n/a (only inh. zone data)	2022	[218]
		Extract from *Yucca gigantea*	14.8	2022	[219]
Pseudomonas	*P. aeruginosa*	Extract from *Scabiosa hymettia*	n/a (only inh. zone data)	2008	[209]
		Extract from *Labisa pumila* Benth	n/a (only inh. zone data)	2011	[211]
		Extract from *Bryophyllum pinnatum* (Lank.) Oken	Various depending on extract	2012	[221]
		Extract from *Kalanchoe fedtschenkoi*	256	2019	[96]
		Extract from *Bupleurum chinense*	n/a	2020	[172]
		Extract from *Yucca gigantea*	10.2	2022	[219]
Salmonella	*S. typhi, S. typhimurium*	Extract from *Bryophyllum pinnatum* (Lank.) Oken	Various depending on extract	2012	[221]
		Extract from *Uapaca heudelotti*	12.5	2020	[212]
		Extract from *Yucca gigantea*	10.5	2022	[219]
Vibrio	*V. cholerae*	Extract from *Combretum erythrophyllum*	n/a	2004	[140]
Gram-Positive Bacteria					
Enterococcus	*E. faecium, E. faecalis*	Extract from *Combretum erythrophyllum*	n/a	2004	[140]
		Pure kaempferol-3,7-O-α-L-dirhamnoside	0.5	2006	[214]
		Extract from *Laurus nobilis*	>256	2008	[222]
Micrococcus	*M. luteus*	Extract from *Labisa pumila* Benth	Various depending on extract	2011	[211]
Staphylococcus	*S. aureus, S. epidermidis*	Extract from *Mitracarpus scaber*	125	2000	[223]
		Pure kaempferol-3,7-O-α-L-dirhamnoside	0.5	2006	[214]
		Extract from *Scabiosa hymettia*	n/a (only inh. zone data)	2008	[209]
		Extract from *Laurus nobilis*	>256	2008	[222]
		Extract from *Allium ursinum*	625	2009	[224]
		Extract from *Platanus occidentalis*	Various depending on the kaempferol compound	2009	[225]
		Extract from *Laurus nobilis*	Various depending on synergistic effects	2009	[206]
		Extract from *Labisa pumila* Benth	Various depending on extract	2011	[211]
		Extract from *Bryophyllum pinnatum* (Lank.) Oken	Various depending on extract	2012	[221]
		Extract from *Calliandra tergemina* (L.) Benth	Various depending on extract type and compound	2014	[226]
		Extract from *Platanus occidentalis*	n/a	2015	[227]
		Extract from *Kalanchoe fedtschenkoi*	256	2019	[96]
		Extract from *Platanus occidentalis*	16	2020	[228]
		Extract from *Bupleurum chinense*	n/a	2020	[172]
		Extract from *Uapaca heudelotti*	12.5	2020	[212]
		Conjugation of pure kaempferol with Ag nanoparticles	n/a	2021	[217]
		Extracts from propolis	n/a—expressed as % of propolis	2021	[216]
		Artificial blend with nanocrystals	n/a—no adm. of sole kaempferol	2021	[215]
		Extract from Yucca gigantea	14.46	2022	[219]
Streptococcus	*S. pyogenes*	Extract from *Uapaca heudelotti*	6.25	2020	[212]
Bacillus	*B. subtilis*, *B. cereus*	Pure kaempferol-3,7-O-α-L-dirhamnoside	8	2006	[214]
		Extract from *Labisa pumila* Benth	Various depending on extract	2011	[211]
		Extract from *Bupleurum chinense*	n/a	2020	[172]
		Extract from *Uapaca heudelotti*	6.25	2020	[212]
		Extract from *Buddleja indica* Lam.	0.48	2021	[167]
		Extract from *Astragalus creticus*	n/a (expr. as inhibition %)	2021	[100]
		Conjugation of pure kaempferol with Ag nanoparticles	n/a	2021	[217]
		Extract from *Rosa damascena* Mill var. *trigintipetala*	n/a (only inh. zone data)	2022	[218]
Cutibacterium	*C. acnes*	Extract from *Impatiens balsamina*	32–64	2007	[208]
Ziehl-Neelsen Stain					
Mycobacterium	*M. bovis*, *M. tuberculosis*	Extract from *Argyreia speciosa*	25	2009	[220]
		Extract from *Doliocarpus dentatus*	62.5	2017	[229]
		Extract from *Pluchea indica*	n/a	2020	[180]
		Extract from *Bauhinia vahlii*	n/a (expr. as inhibition %)	2021	[102]
		Extract from *Vatairea macrocarpa*	n/a	2021	[230]

**Table 4:** Kaempferol-containing extracts and compounds with a verified antifungal potential.

Genus	Species	Tested Substance	MIC (μg/mL)	Year of Research	Reference
Aspergillus	*A. fumigatus*	Extract from *Rosa damascena* Mill var. *trigintipetala*	n/a (only inh. zone data)	2022	[218]
Candida	*C. albicans*, *C. tropicalis*, *C. glabrata*	Extract from *Mitracarpus scaber*	250–500	2000	[223]
		Pure kaempferol-3-O-(6”-galloyl)-β-D-glucopyranoside	200	2004	[342]
		Extract from *Scabiosa hymettia*	n/a (only inh. zone data)	2008	[209]
		Pure kaempferol	25	2008	[343]
		Extract from *Allium ursinum*	>625	2009	[224]
		Extract from *Labisa pumila* Benth	Various depending on extract	2011	[211]
		Extract from *Bryophyllum pinnatum* (Lank.) Oken	Various depending on extract	2012	[221]
		Extract from *Geranium* *ibericum* subsp. *jubatum*	400	2021	[132]
		Extract from *Rosa damascena* Mill var. *trigintipetala*	n/a (only inh. zone data)	2022	[218]
		Bark extract from *Spondias mombin*	n/a	2022	[213]
Cryptococcus	*C. neoformans*	Extract from *Bryophyllum pinnatum* (Lank.) Oken	Various depending on extract	2012	[221]

**Table 5:** Protozoal diseases discussed in the text and their causative agents.

Disease	Causative Agents	Endemic Areas	References
Amoebiasis	*Entamoeba histolytica*	Central and South America, Africa, India	[346,347]
Giardiasis	*Giardia lamblia*	Worldwide	[348,349]
Human African trypanosomiasis	*Trypanosoma brucei gambiense*, *Trypanosoma brucei rhodensiense*, *Trypanosoma brucei brucei*, *Trypanosoma congolense*, *Trypanosoma evansi*	Sub-Saharan (Central and West Africa)	[350]
Malaria	*Plasmodium malariae*, *Plasmodium falciparum*, *Plasmodium vivax*, *Plasmodium ovale*, *Plasmodium knowlesi*	South America, Africa, India, and South Pacific islands	[351,352]
Leishmaniasis	*Leishmania donovani*, *Leishmania major*, *Leishmania mexicana*, *Leishmania tropica*, etc.	Africa, Central and South Asia, Central and South America	[353,354,355]

**Table 6:** Antiprotozoal activity of kaempferol compounds based on current research.

Active Kaempferol Compound	Extracted from	Active against	IC50 (μg/mL)	Year of Study	References
Kaempferol	*Helianthemum glomeratum*	*E. histolytica*	9.7	1995	[136]
Kaempferol	*Helianthemum glomeratum*	*E. histolytica*, *G. lamblia*	7.93, 8.73	1998	[356]
Kaempferol	*Helianthemum glomeratum*	*E. histolytica*, *G. lamblia*	7.93, 8.73	1999	[357]
Kaempferol	*Helianthemum glomeratum*	*E. histolytica*, *G. lamblia*	7.93, 8.73	1999	[358]
Kaempferol-7-methylether	*Alomia myriadenia*	*T. cruzi*	n/a (expressed as % of *T. cruzi* lysis)	2003	[359]
Kaempferol	*Cuphea pinetorum*	*E. histolytica*, *G. lamblia*	7.9–8.3	2005	[141]
Kaempferol	*Morinda morindoides*	*E. histolytica*	Various depending on kaempferol compound	2006	[160]
Kaempferol-3-O-α-L-arabinopyranosyl (1→2)-α-L-rhamnopyranoside	*Kalanchoe pinnata*	*Leishmania* spp.	>100	2006	[360]
8-(1;1)-DMA-kaempferide	Pure compound	*P. falciparum*	n/a (expressed in μΜ)	2006	[361]
Kaempferol	*Eupatorium perfoliatum* L.	*P. falciparum*	2.7 (whole extract)	2011	[179]
Kaempferol	*Cayratia trifolia* Linn	*Trypanosoma* spp.	n/a	2011	[98]
Kaempferol rhamnosides and glycosides	Pure compounds	*Plasmidium* spp.	n/a (expressed in μΜ)	2016	[362]
Kaempferol	*Annona cherimola* Miller	*E. histolytica*, *G. lamblia*	7.9, 8.7	2017	[85]
Kaempferol-3,7-dimethylether	*Cnidoscolus chayamansa*	*E. histolytica*, *G. lamblia*	≤27.43	2017	[363]
Kaempferol-3,7-di-O-methylether	*Solanum paludosum* Moric	*L. amazonensis*	n/a (expressed in μΜ)	2019	[364]
Kaempferol, 4′-methoxykaempferol, 4′,7-dimethoxykaempferol	Propolis	*T. brucei*, *L. mexicana*	n/a (expressed in μΜ)	2021	[365]
Kaempferol, kaempferol 3-O-α-L-rhamnoside, and other kaempferol compounds	*Lotus corniculatus* L.	*Trypanosoma* spp., *Plasmodium* spp.	0.98, 1.57	2021	[366]

**Table 7:** Correlation between ethnobotanical and described uses of certain kaempferol-containing plants.

Plant	Traditional/Ethnobotanical Uses	Uses Described in This Paper	References
*Annona cherimola* Miller	Traditional Mexican medicine	Antiprotozoal (against *E. histolytica*, *G. lamblia*)	[85,395,396]
*Argyreia speciosa*	Traditional Indian medicine	Antibacterial (against *K. pneumoniae*, *M. tuberculosis*)	[220,397,398]
*Astragalus creticus*	Traditional Chinese medicine, traditional Pakistani medicine	Antibacterial (against *B. subtilis*)	[100,399]
*Bauhinia vahlii*	Traditional Indian medicine	Antibacterial (against *M. tuberculosis*)	[102,400,401]
*Bryophyllum pinatum*	Traditional Chinese medicine, various traditional medical systems of tropical Africa and America, traditional Indian medicine	Antibacterial (against *P. aeruginosa*, *S. aureus*, *S. typhi*); antifungal (against *C. neoformans*)	[221,402,403]
*Buddleja indica* Lam	Traditional African medicine	Antibacterial (against *B. subtilis*)	[167,404]
*Bupleurum chinense*	Traditional Chinese medicine	Antibacterial (against *B. subtilis*, *E. coli*, *K. pneumoniae*, *P. aeruginosa*, *S. aureus*, *S. aureus*)	[172,405,406]
*Cnidoscolus chayamansa*	Traditional Mexican medicine	Antiprotozoal (against *E. histolytica*, *G. lamblia*)	[363,407]
*Cuphea pinetorum*	Traditional Mayan medicine, traditional Mexican medicine	Antiprotozoal (against *E. histolytica*, *G. lamblia*)	[141,408]
*Doliocarpus dentatus*	Traditional Brazilian medicine, traditional Peruvian medicine	Antibacterial (against *M. tuberculosis*)	[229,409]
*Geranium ibericum jubatum*	Ethnobotanical usage in Malaya, Eastern Anatolia	Antibacterial (against *A. baumannii*, *K. pneumoniae*)	[132,410]
*Helianthemum glomeratum*	Traditional Mayan medicine	Antiprotozoal (against *E. histolytica*, *G. lamblia*)	[358,411]
*Impatiens balsamina*	Traditional Chinese medicine and traditional medicinal systems of Asia	Antibacterial (against *P. acnes*)	[208,412,413]
*Kalanchoe fedtschenkoi*	Indian traditional medicine, traditional Chinese medicine, traditional Brazilian medicine, Traditional African medicine	Antibacterial (against *A. baumannii*, *P. aeruginosa*, *S. aureus*)	[96,414]
*Labisia pumila* Benth	Traditional Malayan medicine	Antibacterial (against *B. cereus*, *B. subtilis*, *E. aerogenes*, *E. coli*, *K. pneumoniae*, *M. luteus*, *P. aeruginosa*, *S. aureus*); antifungal (against *C. albicans*)	[211,415,416]
*Lotus corniculatus* L.	Traditional Turkish medicine, traditional Russian medicine, traditional Egyptian medicine	Antiprotozoal (against *Trypanosoma* spp.)	[366,417,418]
*Mitracarpus scaber*	Traditional Malian medicine	Antibacterial (against *S. aureus*; antifungal (against *A. fumigatus*)	[223,419]
*Morinda morindoides*	Various traditional African medical systems	Antiprotozoal (against *E. histolytica*)	[160,420,421]
*Pluchea indica*	Traditional Thai medicine, traditional Indian medicine	Antibacterial (against *M. tuberculosis*)	[180,422,423]
Propolis	Ancient Greek medicine, Ancient Roman medicine, Ancient Egyptian medicine, European medieval medical systems	Antibacterial (against *A. baumannii*, *E. coli*, *S. aureus*); antiprotozoan (against *T. brucei*, *L. mexicana*)	[216,365,424]
*Rosa damascena* Mill var. *trigintipetala*	Ancient Persian medicine, traditional Arab medicine, traditional Iranian medicine	Antibacterial (against *B. subtilis*, *E. coli*, *P. vulgaris*); antifungal (*A. fumigatus*, *C. albicans*)	[218,425,426,427,428]
*Scabiosa hymettia*	Traditional Greek medicine	Antibacterial (against *E. coli*, *K. pneumoniae*, *P. aeruginosa*, *S. aureus*, *S. epidermidis*, *E. cloacae*); antifungal (*C. albicans*, *C. glabrata*, *C. tropicalis*)	[209,429]
*Solanum paludosum* Moric	South American folk medicine	Antiprotozoal (against *L. amazonensis*)	[364,430]
*Syzygium aromaticum*	Various traditional medicinal systems of Asia	Antibacterial (against *P. gingivalis*, *P. intermedia*)	[144,431]
*Uapaca heudelotii*	Traditional Congolese medicine, various other local African medical traditions	Antibacterial (against *S. pneumoniae*, *S. aureus*, *S. typhi*, *P. mirabilis*, *B. subtilis*)	[212,432,433]
*Uapaca heudelotti*	Traditional African medicine	Antibacterial (against *B. subtilis*, *S. aureus*, *S. pneumoniae, S. typhi*)	[212,433]
*Vatairea macrocarpa* (Benth) Ducke	Traditional Brazilian medicine	Antibacterial (against *M. bovis*)	[230,434]
*Yucca gigantea*	Native American medicine, traditional Guatemalan medicine	Antibacterial (against *E. coli*, *K. pneumoniae*, *P. aeruginosa*, *S. aureus*, *S. epidermidis*, *S. typhimurium*, *P. mirabilis*)	[219,435,436]

## Data Availability

Not applicable.
